# Funnel-based antimicrobial resistance monitoring in Italy: the FUN-IT study

**DOI:** 10.1038/s41598-025-24383-z

**Published:** 2025-11-18

**Authors:** Simone Milanesi, Marta Colaneri, Sara Laura Ferrari, Alice Baratelli, Simone Villa, Elena Maria Tosca, Pier Mario Perrone, Andrea Gori, Mario Raviglione, Giuseppe De Nicolao

**Affiliations:** 1 Department of Electrical, Computer and Biomedical Engineering, University of Pavia, Pavia, Italy; 2 Department of Biomedical and Clinical Sciences, University of Milan, Milan, Italy; 3 Infectious Diseases Unit II, “L. Sacco” University Hospital, ASST Fatebenefratelli Sacco, Milan, Italy; 4 Centre for Multidisciplinary Research in Health Science (MACH), University of Milan, Milan, Italy; 5 Department of Biomedical Sciences for Health, University of Milan, Milan, Italy; 6 Department of Pathophysiology and Transplantation, University of Milan, Milan, Italy; 7 Division of Infectious Diseases I, Fondazione IRCCS Policlinico San Matteo, Pavia, Italy

**Keywords:** Infectious diseases, Epidemiology

## Abstract

Italy reports some of the highest antimicrobial resistance (AMR) rates in Europe. This necessitates multiple interventions among which improved surveillance is a key to solutions. Statistical Process Control (SPC) methods may help distinguishing between natural variability and significant regional trends. We applied specifically tailored SPC methods, namely funnel plots, Z-score charts, and chi-squared control charts to the AMR data from the AR-ISS surveillance system (2015–2023), focusing on bloodstream infections. Specifically, we analysed regional and temporal trends of carbapenem-resistant *Klebsiella pneumoniae* (CRKP), third-generation cephalosporin-resistant *Escherichia coli* (3GCephRE), carbapenem-resistant *Acinetobacter* spp. (CRAS), carbapenem-resistant *Pseudomonas aeruginosa* (CRPA), vancomycin-resistant *Enterococcus* faecium (VR*E-faecium*), and *Staphylococcus aureus* methicillin-resistant (MRSA). VRE- *faecium* showed a persistent increase at the national level, while other pathogens exhibited marked regional variability. Funnel plots identified significant outliers, particularly for CRAS and CRKP, with peaks in 2020–2021. These trends align with increased antibiotic use during the COVID-19 pandemic. The chi-squared control chart highlighted widening interregional disparities, possibly indicating an uneven distribution of AMR containment efforts across Italy. SPC methods can help highlighting significant deviations and interregional disparities in AMR trends across Italy. The identification of specific outliers suggests these tools can complement traditional surveillance approaches by flagging patterns that may warrant further investigation, supporting targeted public health interventions, especially where regional differences are pronounced.

**Supplementary Information:**

The online version contains supplementary material available at 10.1038/s41598-025-24383-z.

## Introduction

Antimicrobial resistance (AMR) represents a significant and growing global health challenge. Despite its often-silent progression, AMR has catastrophic potential, leading to life-threatening complications and substantial mortality worldwide^1^ In 2021, multi-drug-resistant organisms (MDROs) were directly responsible for an estimated 1.14 million deaths and contributed to 4.71 million deaths at a global level, ranking among the top three leading causes^2^. Based on surveillance reports, Italy stands out as one of the European countries with the highest burden of bacterial AMR^3^, prompting urgent interventions to address AMR and mitigate its long-term public health impact.

The robust surveillance is a cornerstone of AMR mitigation, and its priority is underscored by international frameworks like the Sustainable Development Goals (SDG)^4^, specifically target 3.d.2, which is monitored via the SDG 3.d.2 indicator. This indicator emphasizes the capacity of countries to address health emergencies, including AMR, through standardized reporting. This is made possible by the Global Antimicrobial Resistance and Use Surveillance System (GLASS), a WHO initiative launched in 2015 that provides a unified framework for measuring and reporting AMR, serving as a key platform for tracking global progresses^5^.

Italy contributes to EARS-Net, the European surveillance network, through the National Institute of Health (ISS), which oversees AMR surveillance via the antibiotic-resistance surveillance system coordinated by ISS (AR-ISS). Through its participation in EARS-Net, Italy subsequently contributes to GLASS, the Global Antimicrobial Resistance Surveillance System, thus ensuring its data is integrated into global efforts to monitor AMR. This Italian sentinel network, involving 197 clinical microbiology laboratories across all regions, covers 65.8% of national hospital days (2023) and provides valuable data (antibiotic-resistance percentage) on key pathogen-antibiotic combinations from invasive (blood and cerebrospinal fluid) infections.

Extracting the maximum information from surveillance data requires not only collecting robust epidemiological indicators but also choosing appropriate analytical frameworks. Indeed, traditional approaches to AMR monitoring, such as displaying raw %AMR using boxplots, choropleth maps, and time series^6^, tend to overlook the impact of sample size, potentially confounding natural variability and significant epidemiological events.

Statistical process control (SPC) methods have gained increasing traction in healthcare monitoring since the early 2000s^7–10^ offering a robust framework to analyse and manage natural process variability. i.e., the ensemble of natural fluctuations in a process or phenomenon that occur in the absence of external interventions.

Although funnel plots have been used pioneeringly for a critical analysis of year-on-year reduction targets set by the UK National Health Systems for hospital rates of infection with methicillin resistant *Staphylococcus aureus* (MRSA)^11,12^ there has not been a systematic use of funnel plots for AMR surveillance.

Our study advocates the use of multiple SPC control charts, extending their adoption beyond hospital performance metrics to national-level epidemiological monitoring. This methodology enables both cross-sectional comparison of units (regions) and longitudinal assessment of trends. Furthermore, it offers tools to differentiate outlier behavior such as localized issues (e.g., epidemiological outbreaks, systemic shifts or problems in data collection) from systemic changes (e.g., those observed during the COVID-19 pandemic).

## Methods

### Data

Blood and liquor culture data, aggregated by year, region, pathogen, and main resistance profile, were retrieved from published documents of the National Surveillance on AMR (available at the ISS website^13^. These data correspond to those sent individually to ECDC as part of the EARS-Net surveillance and eventually transferred to WHO GLASS. Since 2024, the National Surveillance on AMR has also collected data on urine cultures, which were not included in this analysis. Specifically, we used data from 2015 to 2023 on MRSA, Vancomycin-resistant Enterococcus faecium (VRE-faecium), third-generation cephalosporin-resistant Escherichia coli (3GCephRE), Carbapenem-resistant Klebsiella pneumoniae (CRKP), Carbapenem-resistant Pseudomonas aeruginosa (CRPA), and Carbapenem-resistant Acinetobacter spp. (CRAS).

No preprocessing has been necessary.

### Funnel-based and multivariate chart control methods

In a funnel plot, a measured or estimated quantity is plotted against an interpretable measure of its precision. A funnel plot is composed of four elements^7^: (i) an indicator $\:Y$ that represents the quantity to be monitored, (ii) a reference value $\:\theta\:$ that specifies the expected value of the indicator, (iii) a precision parameter $\:\rho\:$ that determines the accuracy with which the indicator is measured, (iv) the control limits $\:{y}_{lower}$, $\:{y}_{upper}$ that specify the boundaries of the out-of-control region. An example of funnel plot can be seen in Fig. 2. The point $\:{(\rho\:}_{i},{y}_{i}),\:i=1,\:\dots\:,n$, is associated with the $\:i$-th Italian region, where $\:{\rho\:}_{i}\:$reflects the number of Antimicrobial Susceptibility Tests (ASTs) in the region and $\:{y}_{i}\:$is an AMR indicator. The horizontal centerline $\:Y=\theta\:$ shows the expected value of the indicator and the funnel-shaped pair of control limits $\:{y}_{lower}$ and $\:{y}_{upper}$ show where we would expect the region indicators to be if they were homogenous with those of the population.

In several circumstances, an exact or approximate normal distribution of the indicator $\:Y$ can be assumed, namely

1 $$\:Y\:\sim\:N\left(\theta\:,\frac{g\left(\theta\:\right)}{\rho\:}\right)\:\:\:\:\:\:\:\:\:\:\:\:\:\:\:\:\:\:\:\:\:\:\:$$

where $\:g$ is a suitable function of $\:\theta\:$^7^ such that $\:Var\left[Y\right]=g\left(\theta\:\right)/\rho\:$. Under this null hypothesis, with probability $\:1-\alpha\:$,

$$\:\theta\:-{z}_{\frac{\alpha\:}{2}}\sqrt{\frac{g\left(\theta\:\right)}{\rho\:}}\:\le\:\:Y\:\le\:\:\theta\:+{z}_{\frac{\alpha\:}{2}}\:\sqrt{\frac{g\left(\theta\:\right)}{\rho\:}}$$

where $\:{z}_{{\upalpha\:}/2}$ is such that $\:P\left(Z\le\:{z}_{{\upalpha\:}/2}\:\right)=1-\frac{\alpha\:}{2}$ for a standard normal variable $\:Z$. For instance,

$\:{z}_{{\upalpha\:}/2}\:=1.96$, when $\:\alpha\:=0.05$ and $\:{z}_{{\upalpha\:}/2}\:=3.09$, when $\:\alpha\:=0.002$. This means that, in $\:100\left(1-\alpha\:\right)\%$ of the cases, $\:Y$ is expected to lie within the lower and upper control limits defined as

$$\:{y}_{lower}=\theta\:-{z}_{\alpha\:/2}\sqrt{g\left(\theta\:\right)/\rho\:}$$

$$\:{y}_{upper}=\theta\:+{z}_{\alpha\:/2}\sqrt{g\left(\theta\:\right)/\rho\:}$$

By introducing the Z-score

$$\:{z}_{i}=\frac{{y}_{i}-\theta\:}{\sqrt{g\left(\theta\:\right)/\rho\:}}$$

we have that $\:P\left({|z}_{i}\right|>{z}_{\alpha\:/2})=\alpha\:$. In Statistical Process Control, the common practice is to select a false alarm probability as small as $\:\alpha\:=0.002$, corresponding to $\:{z}_{\alpha\:/2}=3.09.$ A Z-score whose absolute value is greater than $\:{z}_{\alpha\:/2}$ is said to be *out of (statistical) control* and deemed worthy of study to identify a special cause of variation that explains its departure from the mean. Note that there is a $\:0.2\%$ probability of reporting an out-of-control point when no special cause of variation is actually perturbing the process and the outlier arises by pure chance under common causes of variation.

When the indicators $\:{y}_{i}\:$ measure a frequency of occurrence, it is reasonable to assume a binomial model, with $\:\theta\:$ representing the probability of the event and $\:{\rho\:}_{i}$ the number of surgeries in the $\:i$-th unit. For the binomial model, the variance of $\:{y}_{i}$ is $\:\theta\:(1-\theta\:)/{\rho\:}_{i}$ so that, given $\:\theta\:$, the variance of $\:{y}_{i}\:$is completely specified. For a large enough $\:\rho\:$, the binomial converges to a normal random variable that follows distribution (1) with.

$\:g\left(\theta\:\right)=\theta\:(1-\theta\:)$. Therefore, estimating the mean of $\:{y}_{i}\:$suffices to specify both the centerline and the alarm limits of the funnel plot. However, as discussed in^14^, if one lets the variance be specified by the mean, it very often happens that the fraction of units of analysis that lie outside the ideal alarm limits greatly exceeds the theoretical false positive rate. We will deal with this overdispersion phenomenon^14^ by introducing a multiplicative overdispersion parameter $\phi$ to be estimated from data:

2 $$\:Y\:\sim \:N(\theta \:,\frac{{\phi g\left( {\theta \:} \right)}}{{\rho \:}})$$

The control limits and the Z-scores are redefined accordingly as

$$\:y_{{lower}} = \theta \: - z_{{\alpha \:/2}} \sqrt {\phi g\left( {\theta \:} \right)/\rho \:}$$

$$\:y_{{upper}} = \theta \: + z_{{\alpha \:/2}} \sqrt {\phi g\left( {\theta \:} \right)/\rho \:}$$

$$\:z_{i} = \frac{{y_{i} - \theta \:}}{{\sqrt {\phi g\left( {\theta \:} \right)/\rho _{i} } }}$$

Details about parameter estimation can be found in the Supplementary Material.

### Choice of the indicator

Funnel plots are usually used to monitor performance over a given time window. In a surveillance context, however, it makes sense to monitor variation, see e.g^14^., where the ratio between successive performances is examined. Here we consider differences $\:{\stackrel{\sim}{Y}}_{t}={Y}_{t}-{Y}_{t-1}$ between AMRs in consecutive years.

In particular, under a binomial model for infections, yielding a normal approximation, if we assume that $\:Y_{t} \sim N(\theta _{t} ,\theta _{t} \left( {1 - \theta _{t} } \right)/\rho \:_{t} )$, we have that $\tilde{Y}_{t} \sim N\left( {\theta _{t} - \theta _{{t - 1}} ,\tau _{t}^{2} } \right)$, where

$$\:{\tau\:}_{t}^{2}={\theta\:}_{t}\left(1-{\theta\:}_{t}\right)/{\rho\:}_{t}+{\theta\:}_{t-1}\left(1-{\theta\:}_{t-1}\right)/{\rho\:}_{t-1}$$

Letting $\:{\beta\:}_{t}^{2}=({\theta\:}_{t}\left(1-{\theta\:}_{t}\right)+{\theta\:}_{t-1}\left(1-{\theta\:}_{t-1}\right))/2$, the variance $\:{\tau\:}_{t}^{2}$ can be approximated as

$$\:{\tau\:}_{t}^{2}={\beta\:}_{t}^{2}\left(\frac{1}{{\rho\:}_{t}}+\frac{1}{{\rho\:}_{t-1}}\right)=\frac{2{\beta\:}_{t}^{2}}{{\stackrel{-}{\rho\:}}_{t}}$$

where $\:{\stackrel{-}{\rho\:}}_{t}=2{\left[\frac{1}{{\rho\:}_{t}}+\frac{1}{{\rho\:}_{t-1}}\right]}^{-1}$is the harmonic mean of the AST numbers.

In conclusion, we assume that

$$\tilde{Y}_{t} \sim N\left( {\bar{\theta }_{t} ,\frac{{2\beta _{t}^{2} }}{{\bar{\rho }_{t} }}} \right)$$

where $\:{\stackrel{-}{\theta\:}}_{t}={\theta\:}_{t}-{\theta\:}_{t-1}$. Comparing with (1), it is possible to monitor the AMR differences through a funnel plot. Just as in the standard case discussed by Spiegelhalter, control limits computed according to the ideal model will usually result in a disproportionate number of out of control points. Therefore, the final model accounts for an overdispersion factor $\:\varphi _{t}$:

$$\tilde{Y}_{t} \sim N\left( {\bar{\theta }_{t} ,\frac{{\sigma _{t}^{2} }}{{\bar{\rho }_{t} }}} \right)$$

where $$\:\sigma _{t}^{2} = 2\varphi _{t} \beta _{t}^{2}$$.

In summary, the practical implementation of the funnel plot for AMR surveillance explicitly defines all key elements highlighted in the theoretical framework:

i. **Indicator**: we monitor the year-to-year variation in AMR, which captures changes in resistance levels between consecutive years;
ii. **Reference value**: the expected value of the indicator is given by the annual change under the null model, i.e. the country year-to-year change, calculated as the difference between consecutive years of the country %AMR;
iii. **Precision parameter**: the accuracy of the indicator is quantified using the harmonic mean of the annual AST counts;
iv. **Control limits**: boundaries of the out-of-control region are calculated according to the statistical model and by incorporating an overdispersion factor​, ensuring that the funnel plot realistically reflects both the expected variability and the additional variation observed in the data.

### Multivariate control chart

The funnel plot detects out of control units with respect to the reference distribution, highlighting anomalous values with respect to the overall population. Another type of surveillance regards the monitoring of the multivariate distribution of the between year variations of regional AMR’s. This second monitoring aims at identifying systemic changes that affect all regions simultaneously. The most common multivariate control chart, is based on the sum of squares of standardized variates^32^. In particular, the indicator

$$\:{T}_{t}^{2}=\mathop{\sum}\limits_{i=1}^{n}{\left(\frac{{\stackrel{\sim}{Y}}_{t,i}-{\stackrel{-}{\theta\:}}_{t}}{\raisebox{1ex}{${\sigma\:}_{t}$}\!\left/\:\!\raisebox{-1ex}{$\sqrt{{\stackrel{-}{\rho\:}}_{t}}$}\right.}\right)}^{2},\:$$

is approximately distributed as a chi-squared distribution with $\:n-1$ degrees of freedom. Therefore, for each time index, $\:{T}_{t}^{2}$can be plotted against the upper control limit $\:{\chi\:}_{\alpha\:}^{2}$, such that $\:\mathcal{P}\left({\chi\:}_{n-1}^{2}\ge\:{\chi\:}_{\alpha\:}^{2}\right)=\alpha\:$, $\:\alpha\:=0.002$.

### SPC user guide: rationale and tiered approach

To guide the reader, we provide here a step-by-step rationale for the choice and order of the SPC methods adopted. The aim was to build a tiered surveillance toolbox where each chart addresses a specific question on AMR variability and evolution.

1. **Funnel Plot** (regional variability at a given time). Purpose: to define the expected range of natural variability of AMR percentages across regions, accounting for the precision of the estimates (number of AST performed). Strengths: intuitive, widely used in healthcare monitoring, allows to distinguish random fluctuation from unusual regional deviations. Limitations: does not capture temporal dynamics and nationwide systemic shifts.
2. **Z-Score Control Chart** (temporal monitoring of single regions). Purpose: to standardize yearly AMR values and monitor their evolution over time. This chart highlights two critical patterns: (i) shifts, indicating persistent changes, and (ii) rebound effects, indicating transient deviations. Strengths: focuses on within-region time trends, complementary to the funnel plot. Limitations: individual-region perspective; does not detect nationwide systemic shifts.
3. **Multivariate Control Chart** (systemic national changes).*Purpose*: to detect whether changes occur simultaneously across all regions, discriminating between increased heterogeneity and global shifts.*Strengths*: identifies systemic variations that might be overlooked by single-region methods.*Limitations*: does not provide detail on which specific regions are driving the change; requires complementary interpretation with funnel plots.

The tiered use of funnel plots, Z-score charts, and chi-squared charts provides a structured SPC-based toolbox for AMR surveillance. Each tool addresses a different surveillance question (cross-sectional anomalies, longitudinal trends, systemic shifts), and together they enhance interpretability, reproducibility, and practical applicability.

## Results

**Fig. 1:**
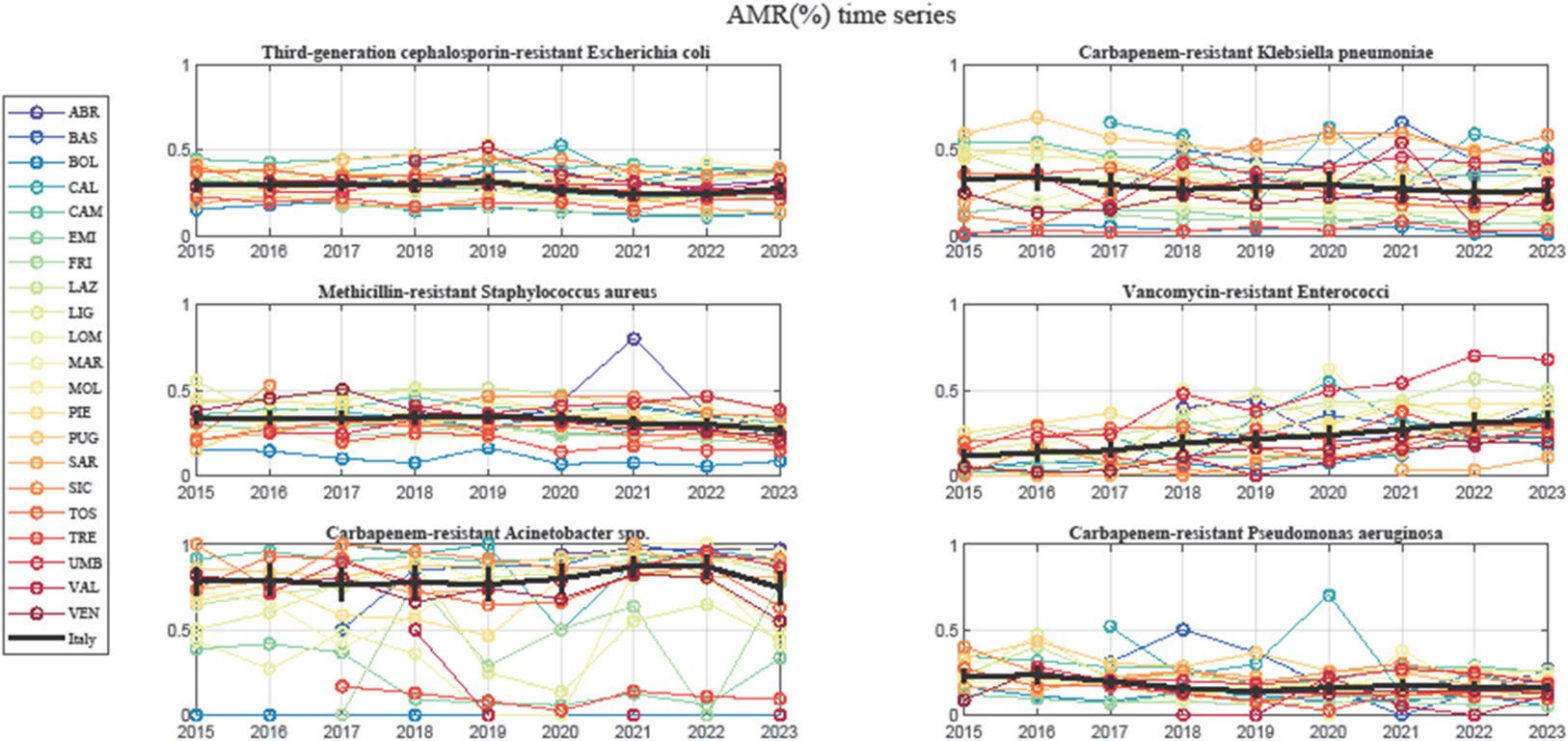
Time series of AMR percentages for six key pathogens in Italy from 2015 to 2023.Each line represents a different region (for full region names, see Supplementary Material), while the bold black line indicates the national average. VRE (Vancomycin-resistant Enterococci) is the only pathogen showing a clear increasing trend at the national level. The remaining pathogens display relatively stable trends. A high degree of heterogeneity is observed across regions, with persistent disparities over time, emphasizing the need for monitoring approaches focused on year-to-year variations.

As seen in Fig. 1, among the six different pathogens, VRE-*faecium* is the only one that shows a clear increasing trend in the average national value. For the other five cases, the national value is relatively stable, maybe with a slight decline for CRKP. Of note is the consistently high value of CRAS.

It is also seen that Italian regional %AMR data are characterized by a significant heterogeneity. Not only regional values are fairly dispersed, but in most cases the differences tend to persist over the years. In view of this, a monitoring approach that focuses on year-to-year variation appears particularly appropriate. In particular, Italian AMR data were analysed by means of the charts of the surveillance toolbox.

### Carbapenem-resistant Acinetobacter spp

**Fig. 2:**
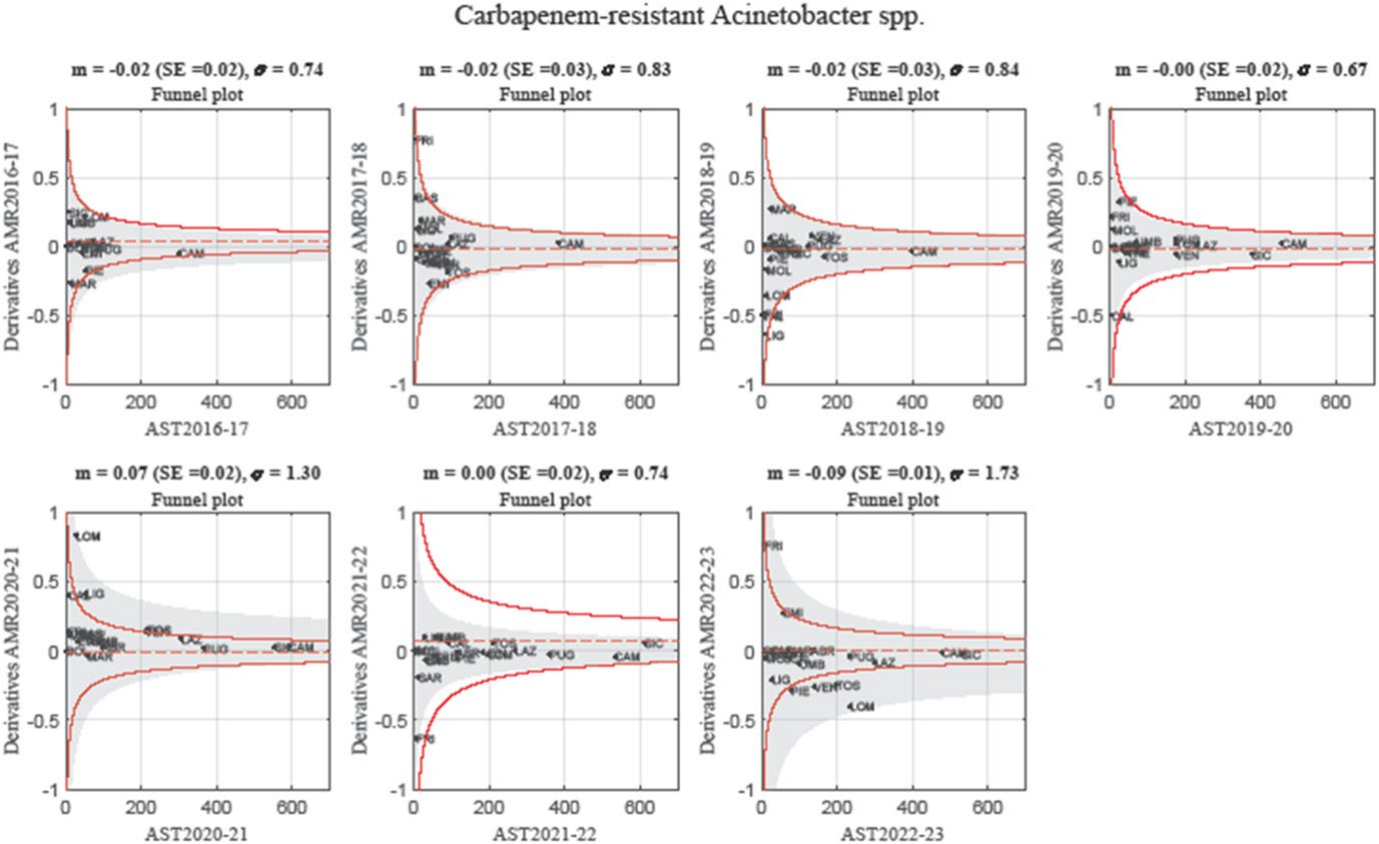
Funnel plots illustrating year-to-year variations in AMR percentage for *Carbapenem-resistant Acinetobacter spp.* (CRAS) across Italian regions, plotted against the harmonic mean of AST counts. Each point represents a regional data value for a specific year. Control limits (red lines) delineate expected variability, with values outside these limits indicating significant deviations. Grey areas represent the current year distribution of the data. Between 2016-2020, all regional data points remained within expected bounds. In 2020-2021, a systemic increase in CRAS prevalence occurred, so that the grey-shaded area expanded beyond the red control limits. A return to statistical stability is observed in 2021-2022, followed by a sharp decline in CRAS resistance in 2022-2023, where Piemonte, Veneto, and Toscana fell below the lower control limits, suggesting potential epidemiological shifts or interventions. Despite this overall reduction, the growing interregional variability observed in 2023 suggests that resistance patterns may be stabilizing at different rates across the country.

Figure 2 presents a series of funnel plots, illustrating the year-to-year variation in %AMR against the harmonic mean of AST counts across Italian regions. Between 2016 and 2017 and 2019–2020, all regional data points remained within the control limits, indicating no significant deviations from expected resistance trends. However, in 2020–2021, the grey-shaded area, representing the current year’s distribution, expanded beyond the upper control limits, suggesting a systemic increase in CRAS prevalence. During this period, Lombardy, Liguria, Veneto, and Tuscany exceeded the upper red margins, highlighting regions with an anomalous increase in AMR derivatives. Lazio approached the threshold but remained within statistical limits.

**Fig. 3:**
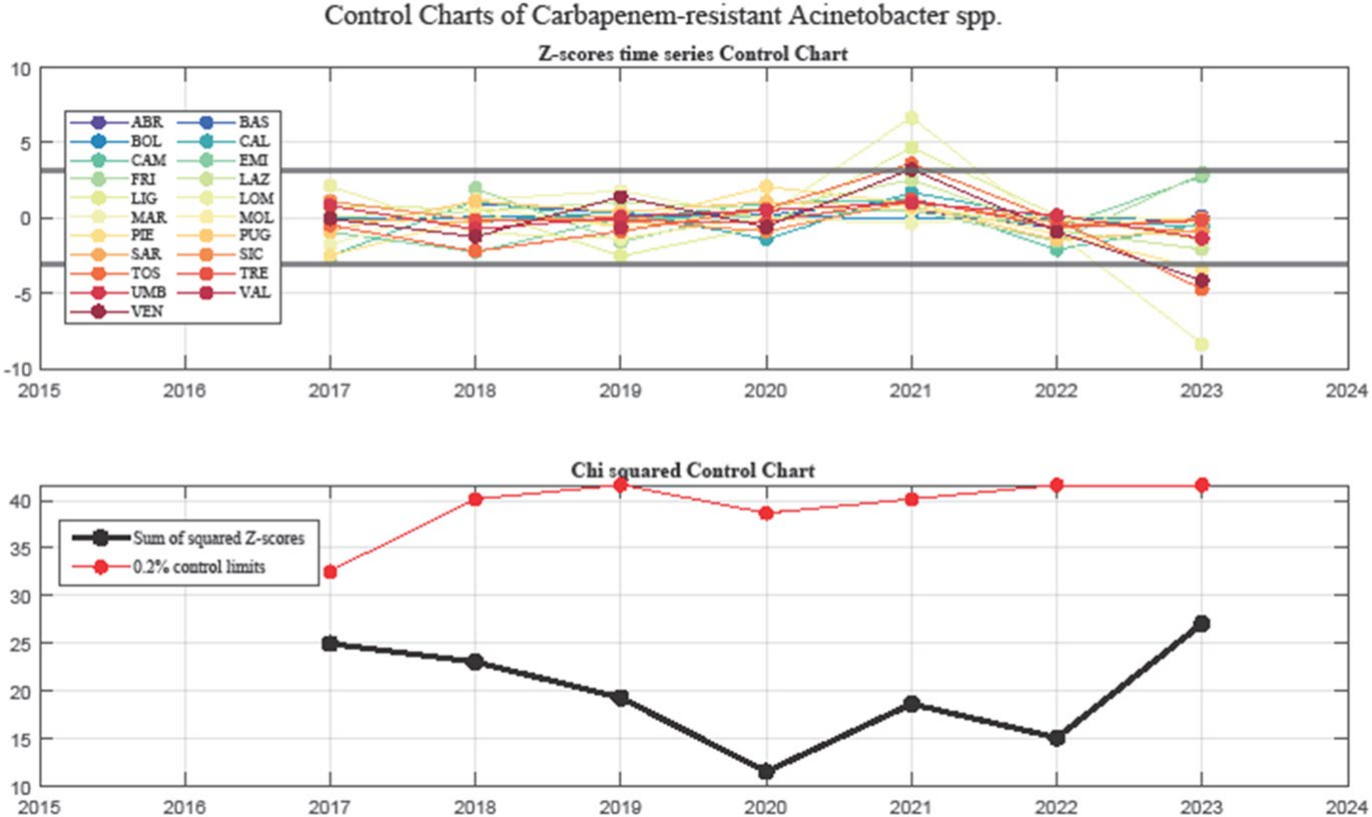
Statistical Process Control charts for CRAS resistance trends in Italy. The top panel presents a Z-score control chart, tracking standardized variations in %AMR across regions over time. Regions exceeding the control limits correspond to outliers identified in the funnel plots. The bottom panel displays a Chi-squared control chart, assessing systemic variation across all regions. A decreasing trend is observed between 2017–2020, followed by a peak in 2023. This trend suggests increasing heterogeneity in regional AMR patterns, highlighting the dynamic nature of resistance evolution and the potential impact of public health interventions.

In 2021–2022, the contraction of the grey area, coupled with all regional data points falling within control limits, suggests a return to statistical stability. However, in 2022–2023, a sharp drop in CRAS resistance was observed in Lombardy, while Piemonte, Veneto, and Tuscany fell below the lower control limit, potentially indicating effective interventions, data biases, or a rebound effect following a previous surge. Figure 3 presents the Z-score control chart (top) and the Chi-squared control chart (bottom). The Z-score chart tracks standardized variations in %AMR over time. Points outside the control limits correspond to the out-of-control regions identified in the funnel plots. The Chi-squared chart (bottom) monitors systemic variations across all regions within control boundaries. A notable decrease in 2017–2020 is followed by a progressive increase from 2020 and a peak in 2023, which may reflect increasing heterogeneity in regional AMR trends.

#### Vancomycin-resistant Enterococcus faecium

**Fig. 4:**
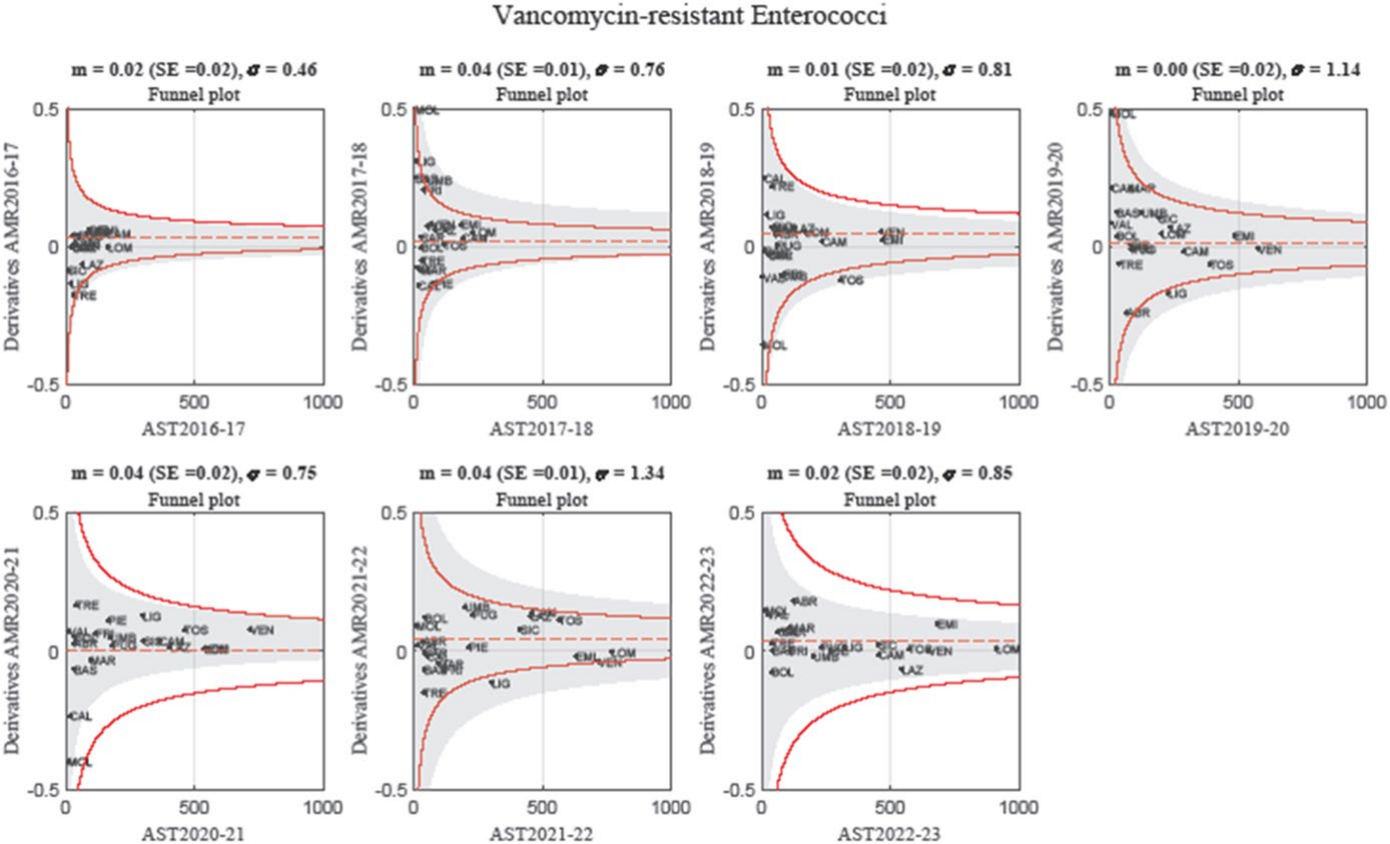
Funnel plots illustrating year-to-year variations in AMR percentage for *Vancomycin- resistant Enterococci* (VRE) across Italian regions, plotted against the harmonic mean of AST counts. In 2016–2017, all regions remained within control limits, indicating stable variations. However, in the following years, certain regions—particularly Molise, Liguria, and Toscana—displayed greater variability. In 2017–2018, Molise and Liguria exhibited the highest resistance values but remained within expected boundaries due to their low AST volumes, whereas Umbria, with a higher AST count, exceeded the upper control limit despite a lower AMR percentage. Toscana showed a notable decline in VRE prevalence in 2018–2019, likely due to successful antimicrobial stewardship (AMS) interventions. In 2020–2021 and 2022–2023, all regions remained within control limits, suggesting a slowdown in the VRE growth. Veneto, Emilia-Romagna, and Lombardia consistently conducted the highest number of ASTs, reflecting strong surveillance capacity.

Figure 4 presents the funnel plots tracking %AMR against the harmonic mean of AST counts for VRE -*faecium*. In 2016–2017, all Italian regions remained within control limits, indicating a stable epidemiological situation. In 2017–2018, Molise and Liguria exhibited the highest resistance variations, yet remained within expected boundaries, likely due to their low AST volumes. Conversely, Umbria, despite having a lower %AMR, exceeded the upper control limit because of a higher AST count, demonstrating the influence of testing volume on statistical thresholds. Between 2018 and 2019, Tuscany showed a decline in VRE -*faecium* prevalence, temporarily moving outside the control limits, a trend that was later mirrored by Liguria in both 2019–2020 and 2021–2022. However, in 2020–2021 and 2022–2023, all regions remained within control limits, suggesting an overall stabilization of VRE-*faecium* trends. Throughout the study period, Veneto, Emilia-Romagna, and Lombardy consistently performed the highest number of ASTs, reflecting their strong surveillance capacity.

**Fig. 5:**
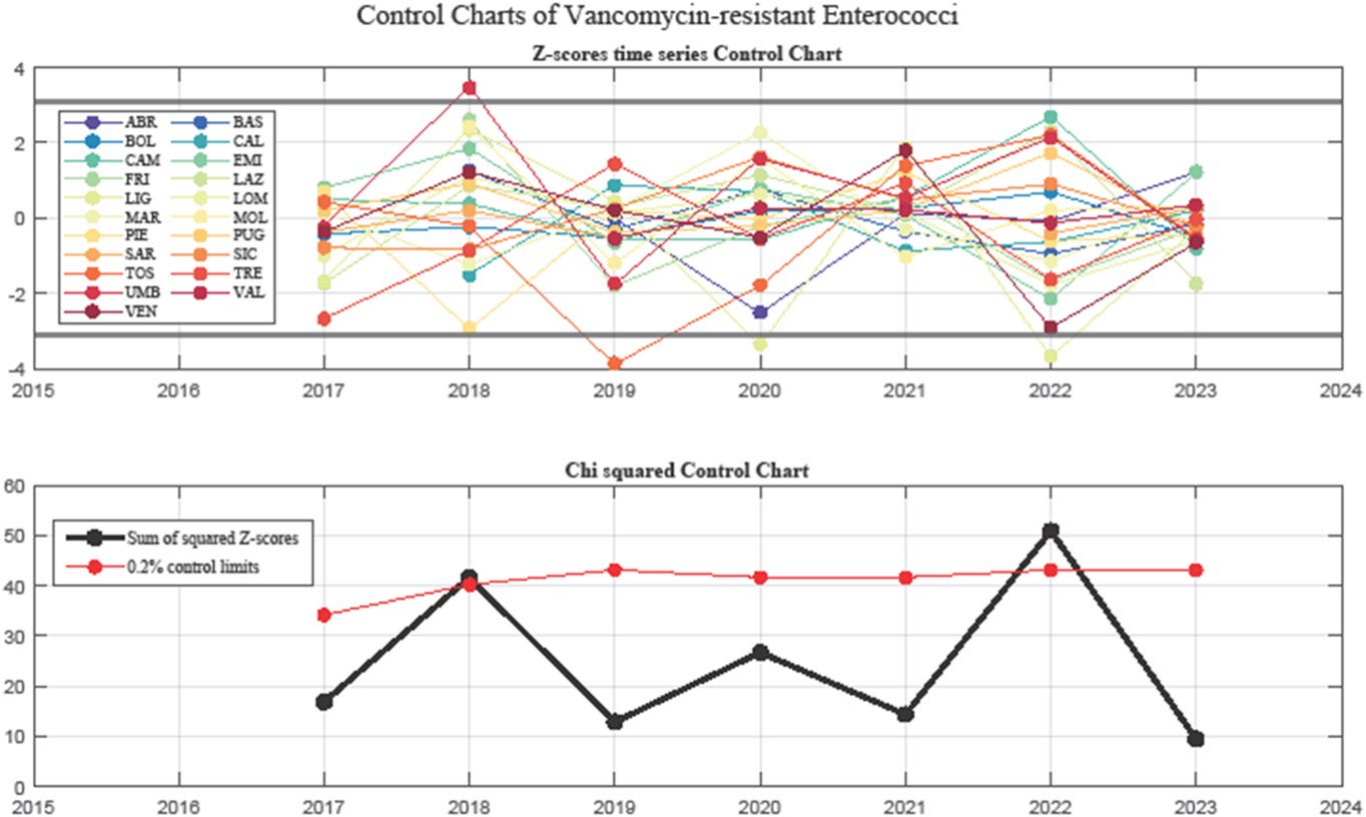
Statistical Process Control charts for VRE resistance trends in Italy. The top panel shows a Z-score control chart, highlighting regional variations over time, in line with the funnel plot findings. The bottom panel presents a Chi-squared control chart, assessing systemic variability. Two major peaks in interregional resistance differences emerged in 2018 and 2022, corresponding to documented outbreaks and changes in infection control policies. Toscana’s decline in VRE prevalence in 2018–2019 aligns with the effects of AMS programs, while Liguria’s persistent resistance trends from 2019 to 2022 suggest localized outbreaks and challenges in infection control. The sharp decline in systemic variability observed in 2023 suggests a decrease in heterogeneity.

Figure 5 presents the Z-score control chart (top) and the Chi-squared control chart (bottom). The top chart provides a temporal overview of the monitoring, highlighting individual variations over the years, consistently with the funnel plots. The Chi-squared control chart monitors systemic variability in VRE-*faecium* resistance: the black cumulative trend line exhibited two major peaks (2018 and 2022), both exceeding control limits, indicating periods of increased interregional variability. In 2023, the trend line sharply declined, suggesting a potential stabilization phase.

#### Carbapenem-resistant Pseudomonas aeruginosa

Figure S1 presents the funnel plots for CRPA. In 2017–2018, Emilia-Romagna approached the upper red limit but remained within control boundaries. By 2018–2019, it returned fully within the limits, while increasing AST volume, indicating enhanced surveillance efforts. In 2019–2020, Tuscany slightly exceeded the upper control limit, although it remained within the grey-shaded area, suggesting a moderate but non-significant increase in CRPA prevalence. From 2020 to 2021 to 2021–2022, all regions remained within statistical thresholds, except for Calabria, which in 2020–2021 fell below the lower control limit, though with a limited AST sample size. In 2022–2023, Lombardy conducted the highest number of ASTs, followed by Tuscany and Emilia-Romagna, both of which approached the upper control limit but remained within expected variability.

The Z-score control chart (top) of Fig. 4 provides a temporal overview of the monitoring, highlighting individual variations over the years, consistently with the funnel plots. The Chi-squared control chart (bottom) remained below control limits but exhibited a transient surge in AMR variability in 2020, followed by a rapid decline in 2021–2022. However, in 2023, the trend rebounded to 2020 levels, suggesting potential increasing heterogeneity in resistance patterns.

#### Third-generation cephalosporin-resistant Escherichia coli

The funnel plots in Figure S3 show a stable resistance pattern from 2016 to 2018, with all regions remaining within control limits. However, in 2018–2019, Lazio exceeded the upper control limits, while Tuscany and Veneto recorded a substantial increase in AST testing. In 2019–2020, the grey area shifted downward and widened, indicating increased variability and an overall reduction in resistance levels. During this period, Lazio fell below the lower control limit, confirming a statistically significant decline. From 2020 to 2023, Emilia-Romagna, Lombardy, Tuscany, and Veneto consistently performed the highest number of ASTs.

In the Z-score control chart (top) of Figure S4, the rebound effect can be observed for Lazio, highlighting possible anomalies in collecting data or an outbreak that rapidly subsided. The Chi-squared control chart highlights an out-of-control value in 2020, corresponding to the global downward shift in the funnel already observed in Figure S2.

#### Carbapenem-resistant Klebsiella pneumoniae

Between 2016 and 2019, all regions remained within control limits (Figure S5). However, in 2019–2020, Sicily exceeded the upper control limits, followed by Lazio in 2021–2022, indicating localized surges in CRKP prevalence. From 2020 to 2023, Lazio, Veneto, Sicily, Tuscany, and Lombardy consistently maintained high AST rates, with Emilia-Romagna performing the highest number of ASTs across all years.

The Chi-squared control chart in Figure S6 remained within statistical control limits throughout the study period, suggesting no major systemic shifts in interregional resistance variability.

#### Methicillin-resistant Staphylococcus aureus

In 2017–2018, Veneto fell below the lower control limit (Figure S7), while Piemonte and Lombardy approached the lower margin, suggesting a temporary decline in resistance rates. In 2018–2019, all regions remained within control limits, with Veneto and Emilia-Romagna showing a notable increase in AST testing, a trend that persisted through 2020–2021. In contrast, 2021–2022 saw Piemonte and Lazio exceed the upper control limit, suggesting a localized increase in resistance prevalence. By 2022–2023, Piemonte exhibited a recovery, falling below the lower red limit, while Lombardy recorded a substantial increase in AST volume.

The Chi-squared control chart in Figure S8 revealed physiological oscillations within statistical limits, aligning with the expected national interregional distribution of MRSA resistance trends.

## Discussion 

This study demonstrates how SPC methods may enhance AMR surveillance by identifying both local anomalies and systemic patterns across regions and over time. This involves two sets of problems. Firstly, the choice of the most suitable SPC tools and, if necessary, the development of new techniques. Secondly, it is crucial to assess in the field whether this paradigm is able to offer added value in terms of national surveillance.

Regarding the first issue, the heterogeneous sample size is a key feature of AMR data. This motivated the use of funnel plots, which were specifically introduced and developed to address statistical challenges associated with varying sample sizes. However, their potential was not fully realized in the context of AMR surveillance.

Furthermore, it is important to acknowledge the structural features of the Italian healthcare system. Since healthcare in Italy is largely managed at the regional and autonomous provincial levels, variations in healthcare practices can influence both the reporting and the management of AMR data. While national guidelines provide a general framework, each region retains discretion over policies, implementation, and reporting. These differences may affect both the quality and the consistency of regional AMR data and should therefore be taken into account when interpreting surveillance results.

Application of funnel plots to AMR surveillance is new apart from an example relative to the assessment of MRSA rates^11^. There, the control limits were based on conditioning on the total observed cases, so that the (approximate) size parameter was given by the average number of cases in consecutive years. In our approach we focus on %AMR using a more insightful size parameter, namely the harmonic mean of ASTs, directly linked to the monitoring effort of the Italian regions.

The regional %AMR provides a straightforward interpretation of resistance levels but relies on the statistical assumption of national homogeneity, which may not hold due to substantial differences in healthcare systems across regions. For this reason, AMR variations are preferred as indicator as they help neutralize disparities, allowing regions to be compared within the same probabilistic framework and highlighting anomalous increases or decreases relative to their own trends.

Building on this methodological framework, we applied SPC techniques to AMR surveillance data across Italian regions, covering six key MDROs. This highlighted both localized anomalies and broader systemic trends observed over the 2015–2023 period.

Regarding CRAS prevalence, while initial years (2016–2020) showed stable resistance within control limits, the pandemic years marked a clear systemic deviation beyond control limits, especially in regions such as Liguria and Lombardy. These findings align with previous studies that documented increased use of broad-spectrum antibiotics and ICU overcrowding during COVID-19, conditions favouring the spread of MDROsCarbapenem resistant *Acinetobacter baummannii* (CRAB)^15^ wordlwide and specifically in Northern Italy^16^. Although CRAB incidence has since decreased^17^, the variability observed underscores the importance of monitoring interregional dynamics rather than relying solely on national aggregates.

Similarly, while initial years showed overall stability of VRE-*faecium*, certain regions, most notably Molise, Liguria, and Tuscany, demonstrated fluctuations over time. These fluctuations in VRE-*faecium* rates in regions like Molise and Liguria might be traced back to localized interventions or shifts in infection control policies. The heightened VRE-*faecium* rates in some regions are potentially attributed to both increased use of vancomycin in ICUs and a shortage of trained infection control staff^18–20^. Tuscany’s and Liguria’s reduction in VRE-*faecium* prevalence from 2018 to 2019 suggests successful interventions in antimicrobial stewardship (AMS) programs^21^ and improved surveillance of potentially colonizing MDROs^22^, respectively. The broader peaks in systemic resistance variability suggest that while regional interventions were effective, they may not have been uniformly implemented across all areas, and notably the funnel plots allowed us to contextualize these events within national trends, identifying both successful interventions and areas where resistance remained persistently high.

In contrast, for MRSA the funnel plot showed remarkable regional stability, with a few notable exceptions such as Veneto falling below the lower control limit in 2017–2018 and Piemonte and Lombardy approaching the lower boundaries. The general downward trend and low variability suggest the effectiveness of long-standing infection prevention efforts and AMS programs, particularly in regions like Veneto, Lombardy, and Piedmont^23–25^. The regional variability in CRPA resistance, especially in regions like Tuscany, reflects trends observed across Europe. Studies have shown that *P. aeruginosa* has been particularly problematic in ICUs during the COVID-19 pandemic^26^.

Reports from Tuscany indicated an increase in CRPA during the early pandemic phase due to factors such as overcrowding in ICUs and the increased use of carbapenems for treating COVID-related pneumonia^27^. Interestingly, systemic monitoring revealed a transient increase in resistance heterogeneity in 2020, followed by a decline in 2021–2022. This suggests that interventions were effective in restoring a more homogeneous situation in the years following the COVID-19 pandemic.

The observed increased incidence of 3GCephRE in Lazio during the pandemic was probably linked to extended use of broad-spectrum antibiotics in hospitals treating high-risk COVID-19 patients^28^. However, by 2020, interventions in AMS programs appeared to have stabilized 3GCephRE rates^29^, as reflected in the return to control limits in later years. This aligns with the broader trend observed in systemic surveillance, where an initial increase in heterogeneity in 2020 was followed by stabilization.

The CRKP results demonstrated relative stability across regions, although some regions like Sicily showed a deviation above control limits in 2019–2020. *K. pneumoniae* resistance in regions such as Sicily has been linked to several outbreaks that emerged in the wake of COVID-19. Specifically, some authors reported an alarming rise in CRKP cases during 2020–2021^30^, likely due to the increased use of carbapenems in COVID-19 patients. By 2023, however, evidence suggested a gradual decline in CRKP rates, which might reflect the effectiveness of national efforts to curb the spread of hospital-acquired infections through improved AMS and infection control programs^31^.

The SPC allowed for the early detection of abnormal trends in AMR rates which is particularly significant since even small shifts in resistance patterns can have serious public health implications. Unlike traditional reports that rely on aggregated data, SPC helps identify specific regions and time periods where AMR deviates from expected norms. This robust analytical approach distinguishes between random fluctuations and genuine deviations, which can signal the need for targeted interventions. For instance, regions like Liguria and Tuscany showed deviations in CRAS and VRE-*faecium*, indicating a need for targeted interventions. Ideally, applying SPC to real-time data could immediately alert public health officials to potential outbreaks, allowing for quicker and more effective interventions. While our retrospective analysis offers valuable insights into past trends, real-time application of SPC might provide a timely and effective response to AMR outbreaks. However, there are some limitations to consider. The retrospective nature of the data means that our findings reflect past trends and might not fully represent current AMR dynamics. Furthermore, while SPC can identify regions and timeframes with significant deviations, it doesn’t provide direct insight into the causes behind those shifts, such as changes in hospital practices, AMS programs, or the introduction of other infection control measures.

It has already been pointed out that the application of SPC in healthcare comes with several practical benefits, limitations, and implementation challenges^32^. Among the main advantages, SPC stands out as a relatively low-cost and versatile tool that can support process improvement across a wide range of clinical and organizational settings. There are also limitations. Displaying data in control chart format does not inherently lead to improved outcomes, and processes deemed to be in statistical control may still fall short of clinical expectations. Barriers to SPC adoption include resistance to change and limited access to high-quality data. To overcome these barriers, training users in SPC principles and providing technical support are essential. Moreover, a common misconception is the belief that the theoretical foundations of SPC are difficult to master. In reality, the theory is entirely based on probability theory and is intuitive: natural variability is modeled using an appropriate probabilistic model that can be adapted to the nature of the data, and control limits are derived accordingly. Another misconception is that normality is required. In fact, other probabilistic distributions can also be used to model the phenomenon. However, normality guarantees specific and useful properties and is recommended when addressing overdispersion.

## Supplementary Information

Below is the link to the electronic supplementary material.

Supplementary Material 1

## Data Availability

The study did not involve humans, and it was conducted in accordance with the local legislation and institutional requirements. The original contributions presented in the study are included in the article/Supplementary material, further inquiries can be directed to the corresponding author Giuseppe De Nicolao (giuseppe.denicolao@unipv.it).
