# Supplementary material for: Funnel-based antimicrobial resistance monitoring in Italy: the FUN-IT study
Source: Sci Rep. 2025 Nov 18;15:40477. doi: 10.1038/s41598-025-24383-z (PMC12627780; doi:10.1038/s41598-025-24383-z)
Supplement: Supplementary file 1 — Supplementary Material 1 [file 41598_2025_24383_MOESM1_ESM.docx]

**SUPPLEMENTARY MATERIAL
Funnel-based Antimicrobial Resistance Monitoring in Italy: the FUN-IT study**

**Glossary of Italian Regions**

For clarity and ease of reference throughout the manuscript, we provide a glossary of Italian regions and their corresponding acronyms used in the AMR surveillance data:

| **Acronym** | **Region/Province** |
| --- | --- |
| ABR | Abruzzo |
| BAS | Basilicata |
| BOL | Bolzano (Provincia Autonoma) |
| CAL | Calabria |
| CAM | Campania |
| EMI | Emilia-Romagna |
| FRI | Friuli Venezia Giulia |
| LAZ | Lazio |
| LIG | Liguria |
| LOM | Lombardia |
| MAR | Marche |
| MOL | Molise |
| PIE | Piemonte |
| PUG | Puglia |
| SAR | Sardegna |
| SIC | Sicilia |
| TOS | Toscana |
| TRE | Trento (Provincia Autonoma) |
| UMB | Umbria |
| VAL | Valle d’Aosta |
| VEN | Veneto |

**Parameter estimation**

Under (2), the *i*-th observation can be written as

$y_{i}= \theta+ \frac{\varepsilon_{i}}{\sqrt{x_{i}}}$ (5)

where $x_{i}=\rho_{i}$ is the size parameter, and $\varepsilon_{i} \sim N(0,\sigma^{2})$, $i=1,\ldots,n$, are mutually independent with

$\sigma^{2}=\phi g(\theta)$. Letting $v_{i}=\varepsilon_{i}/\sqrt{x_{i}}$, the model in matrix form becomes

$$Y=\Phi\theta+v$$

where $Y=[\ldots Y_{i}\ldots]'$, $\Phi=\left[ \ldots1\ldots\right]^{'}$, $v\sim N(0,\sigma^{2}\Sigma),$ and

$$\Sigma=\left( \begin{aligned} \frac{1}{x_{1}} \cdots0 \\ \\ \vdots\ddots\vdots\\ \\ 0 \cdots\frac{1}{x_{n}} \end{aligned} \right)$$

Then, the generalized least squares technique[14] provides the estimates

$$\hat{\theta}=\left( \Phi^{'}\Sigma^{-1}\Phi\right)^{-1}\Phi'\Sigma^{-1}Y$$

$\hat{\sigma^{2}}= \frac{1}{n-1} e^{'}\Sigma^{-1}e$ (6)

where $e=Y-\Phi\hat{\theta}$ is the vector of the residuals.

**Table S1: Missing data for each considered MDRO, classified by year and affected Italian region**

| **MDRO** | **Years with missing data** | **Regions with missing data** |
| --- | --- | --- |
| *Carbapenem-resistant Escherichia coli* | 2016 | ABR, BAS, CAL, FRI, MOL, UMB, VAL |
|  | 2017 | ABR, BAS, CAL, FRI, MOL, VAL |
|  | 2018 | ABR, VAL |
|  | 2020 | SAR |
|  | 2021 | SAR |
| *Carbapenem-resistant Klebsiella pneumoniae* | 2016 | ABR, BAS, CAL, FRI, MOL, UMB, VAL |
|  | 2017 | ABR, BAS, CAL, FRI, MOL, VAL |
|  | 2018 | ABR, VAL |
|  | 2019 | ABR |
|  | 2020 | ABR, SAR |
|  | 2021 | SAR |
| *Methicillin-resistant Staphylococcus aureus* | 2016 | ABR, BAS, CAL, FRI, MOL, TRE, UMB, VAL |
|  | 2017 | ABR, BAS, CAL, FRI, MOL, SIC, TRE, VAL |
|  | 2018 | ABR, SIC, VAL |
|  | 2020 | SAR |
|  | 2021 | SAR |
| *Vancomycin-resistant Enterococci* | 2016 | ABR, BAS, CAL, FRI, MOL, UMB, VAL |
|  | 2017 | ABR, BAS, CAL, FRI, MOL, VAL |
|  | 2018 | ABR, VAL |
|  | 2020 | SAR |
|  | 2021 | SAR |
| *Carbapenem-resistant Acinetobacter spp.* | 2016 | ABR, BAS, CAL, FRI, MOL, TRE, UMB, VAL |
|  | 2017 | ABR, BAS, CAL, FRI, MOL, TRE, VAL |
|  | 2018 | ABR, VAL |
|  | 2019 | ABR |
|  | 2020 | ABR, SAR, VAL |
|  | 2021 | SAR, VAL |
|  | 2022 | VAL |
|  | 2023 | VAL |
| *Carbapenem-resistant Pseudomonas aeruginosa* | 2016 | ABR, BAS, CAL, FRI, MOL, TRE, UMB, VAL |
|  | 2017 | ABR, BAS, CAL, FRI, MOL, TRE, VAL |
|  | 2018 | ABR, VAL |
|  | 2019 | ABR |
|  | 2020 | ABR, SAR |
|  | 2021 | SAR |
| *Note* ABR = Abruzzo, BAS = Basilicata, CAL = Calabria, FRI = Friuli Venezia Giulia, MOL = Molise, SAR = Sardinia, SIC = Sicily, TRE = Trentino-Alto Adige, UMB = Umbria, VAL = Aosta Valley | | |

**Figure S1: Funnel plots illustrating year-to-year variations in AMR percentage for *Carbapenem-resistant Pseudomonas aeruginosa* (CRPA) across Italian regions, plotted against the harmonic mean of AST counts.** In 2017-2018, Emilia-Romagna approached the upper control limit but remained within statistical boundaries. By 2018-2019, it returned fully within limits, coinciding with an increase in AST volume, likely reflecting enhanced surveillance. In 2019-2020, Toscana slightly exceeded the upper control limit but remained within the grey-shaded area, indicating a significant increase in CRPA prevalence that is also consistent with the national behavior. From 2020 to 2022, all regions remained within control limits, except for Calabria in 2020-2021, which fell below the lower boundary, though based on a limited AST sample size. In 2022-2023, Lombardia conducted the highest number of ASTs, while Toscana and Emilia-Romagna approached the upper control limit but remained within expected variability. The regional variability in CRPA resistance, particularly in Toscana, reflects trends observed across Europe. *Pseudomonas aeruginosa* has been a major concern in intensive care units (ICUs), especially during the COVID-19 pandemic, when overcrowding and increased carbapenem use for COVID-related pneumonia contributed to rising resistance levels. Reports from Toscana indicate a surge in CRPA cases during the early pandemic phase, likely driven by these factors.

**Figure S2: Statistical Process Control charts for CRPA resistance trends in Italy.** The Z-score control chart (top) tracks individual regional variations over time, showing that the 2021 out-of-control value for Calabria could be part of a rebound effect following the 2020 increase, i.e. a positive %AMR jump in 2020 followed by a recovery. The Chi-squared control chart (bottom) remained below control limits but showed a transient surge in AMR variability in 2020, followed by a rapid decline in 2021-2022. This pattern suggests that systemic interventions were effective in restoring more homogeneous resistance trends in the years following the COVID-19 pandemic. However, in 2023, the trend rebounded to 2020 levels, indicating a potential increase in regional heterogeneity.

 **Figure S3: Funnel plots illustrating year-to-year variations in AMR percentage for *Carbapenem-Resistant Escherichia coli* (3GCephRE) across Italian regions, plotted against the harmonic mean of AST counts.** From 2016 to 2018, all regions remained within control limits, with Lazio exceeding the upper control limits in 2018-2019. This fluctuation can be attributed to the broader increase in third-generation cephalosporin-resistant organisms in Italian hospitals during this period, largely due to the extended use of broad-spectrum antibiotics in the treatment of high-risk COVID-19 patients. Lazio experienced several 3GCephRE outbreaks following the onset of the pandemic. However, by 2020, regional interventions in antimicrobial stewardship programs helped stabilize 3GCephRE rates, which is reflected in the return to control limits in subsequent years. Toscana and Veneto recorded a significant increase in AST testing. In 2019-2020, a downward shift and widening of the grey area indicated increased variability and reduced resistance levels, with Lazio falling below the lower control limit. From 2020 to 2023, all regions remained within control limits, with Emilia-Romagna, Lombardia, Toscana, and Veneto consistently performing the highest number of ASTs.

 **Figure S4: Statistical Process Control charts for 3GCephRE resistance trends in Italy.** The Z-score chart highlights individual variations and the rebound effect observed in Lazio between 2019 and 2020. The Chi-squared chart reveals an out-of-control value in 2020, corresponding to the global downward shift in resistance levels seen in Figure S3.

 **Figure S5: Funnel plots illustrating year-to-year variations in AMR percentage for *Carbapenem-Resistant Klebsiella pneumoniae* (CRKP) across Italian regions, plotted against the harmonic mean of AST counts.** Between 2016 and 2019, all regions remained within the control limits, reflecting relative stability in CRKP rates across Italy. However, Sicilia exceeded the upper control limits in 2019-2020, followed by Lazio in 2021-2022, indicating localized surges in CRKP prevalence. In Sicilia, these increases were likely associated with outbreaks that surged during the COVID-19 pandemic, driven by the overuse of carbapenems in patients with COVID-19. By 2023, however, evidence suggested a gradual decline in CRKP rates, possibly due to national efforts focused on improving infection control and antimicrobial stewardship.

 **Figure S6: Statistical Process Control charts for CRKP resistance trends in Italy.** The top chart provides a temporal perspective on the monitoring, highlighting individual regional variations over the years, consistent with the trends observed in the funnel plots. The Chi-squared control chart (shown in Figure S5) remained within statistical control limits throughout the study period, suggesting no major systemic shifts in interregional resistance variability. This aligns with the observed decline in CRKP rates by 2023, reflecting the positive impact of ongoing infection control measures and stewardship interventions.

**Figure S7: Funnel plots illustrating year-to-year variations in AMR percentage for *Methicillin-resistant Staphylococcus aureus* (MRSA) across Italian regions, plotted against the harmonic mean of AST counts.** Most regions remained within control limits throughout the study period, indicating overall stability. However, in 2017-2018, Veneto fell below the lower control limit, likely driven by strict hospital hygiene measures and targeted stewardship programs. Piemonte and Lombardia also neared the threshold, suggesting a temporary decline in resistance. In 2018-2019, all regions remained within expected variability, while Veneto and Emilia-Romagna showed a notable increase in AST testing, a trend that continued through 2020-2021. In 2021-2022, Piemonte and Lazio exceeded the upper control limit, indicating a localized rise in resistance. By 2022-2023, Piemonte showed a reversal, dropping below the lower control limit, while Lombardia experienced a significant increase in AST volume.

**Figure S8: Statistical Process Control charts for MRSA resistance trends in Italy.** The Z-score chart highlights regional variations, notably the rebound effect in Piemonte, where %AMR exceeded the upper limit in 2022 before returning to lower levels in 2023. A similar pattern, though within limits, is observed in Lazio. In the Chi Squared control chart, the observed fluctuations remained within statistical limits, reflecting expected interregional variations and overall consistency in national resistance patterns.
